# Influence of midazolam-related genetic polymorphism on conscious sedation during upper gastrointestinal endoscopy in a Korean population

**DOI:** 10.1038/s41598-019-52517-7

**Published:** 2019-11-05

**Authors:** Jae Yong Park, Beom Jin Kim, Sang Wook Lee, Hyun Kang, Jeong Wook Kim, In-Jin Jang, Jae Gyu Kim

**Affiliations:** 1 Department of Internal Medicine, Chung-Ang University College of Medicine, Seoul, Korea; 2 Department of Anestheology and Pain Medicine, Chung-Ang University College of Medicine, Seoul, Korea; 3 Department of Clinical Pharmacology and Therapeutics, Seoul National University College of Medicine and Hospital, Seoul, Korea

**Keywords:** Outcomes research, Oesophagogastroscopy

## Abstract

Genetic polymorphism can result in abnormal pharmacodynamics that subsequently leads to the individual variance in sedative effects and adverse reactions. The aim of this study was to elucidate the association between midazolam-related genetic polymorphism and sedative effects, including adverse reactions, under conscious sedation during upper gastrointestinal endoscopy. We prospectively enrolled 100 eligible patients undergoing upper gastrointestinal endoscopy. The efficacy of the sedation, adverse reactions, plasma concentration of midazolam and 1-hydroxymidazolam were investigated as well as the genetic polymorphism of *MDR1* and *CYP3A5*. The correlation between genetic polymorphism and sedative effects was assessed. Regarding *MDR1* gene, the plasma concentration of midazolam was greater in patients with CGC haplotype (*P* = 0.012), while it was lower in patients with CAC haplotype (*P* = 0.005) than in those with other haplotypes. However, genetic polymorphism of neither *MDR1* nor *CYP3A5* correlated with the plasma concentration of 1-hydroxymidazolam. CGT haplotype of *MDR1* was significantly correlated with sedation grade after midazolam administration (*P* = 0.042). In contrast, genetic polymorphism of *CYP3A5* was not correlated with sedation grade. There was no association between genetic polymorphism of *MDR1* or *CYP3A5* and selected adverse reactions related to midazolam. Genetic polymorphism of *MDR1* influences the concentration of midazolam and the sedation grade. However, it is not associated with adverse reactions such as paradoxical response and retrograde amnesia.

## Introduction

Upper gastrointestinal (GI) endoscopy is a common and essential procedure performed for diagnostic or therapeutic purposes in patients with GI symptoms^1,2^. The advancement of endoscopic technique and expansion of its application area was largely attributable not only to the development of the endoscopic device itself, but also to the improvement of sedation methods and drugs^3^. Conscious sedation in GI endoscopy was introduced to promote the patient tolerance and cooperation during the procedure^4^. Sedation can reduce the patient’s fear of the procedure through anxiolytic effect and transient amnesia which allows the patients to undergo an endoscopy in a comfortable state.

To achieve periprocedural safety and patient compliance, sedative drugs should lead to rapid recovery after endoscopic procedures, minimizing the occurrence of adverse reactions. Among various types of sedative drugs available, midazolam is the most widely used drug for conscious sedation in GI endoscopy^5^. This is due to its several advantages such as a short half-life, fast onset of sedation, and strong anterograde amnesia effect^6^. However, as the effective dose range is considerably variable among individuals, this might cause difficulties in achieving stable sedation during the procedure as well as oversedation-related problems, such as respiratory depression or resedation.

Predictive factors of adverse reactions to midazolam during GI endoscopy, including paradoxical response and retrograde amnesia, are unclear^7^. Furthermore, there is an absence of a well-defined set of practice guidelines for the determination of patient suitability for successful conscious sedation in GI endoscopy^5^. A recent study reported that patients with genetic variability in the gamma-aminobutyric acid (GABA) receptor may result in an abnormal pharmacodynamic response including a differing affinity for midazolam^8^.

Therefore, we hypothesized that genetic polymorphism can alter the pharmacodynamics of midazolam, eventually inducing variances in sedative effects and adverse reactions. The aim of this study was to elucidate the association between midazolam-related genetic polymorphism and sedative effects, including adverse reactions, under conscious sedation during upper GI endoscopy.

## Methods

### Study design and patient selection

This was a prospective observational study, and patients aged 18–70 years who underwent upper GI endoscopy at Chung-Ang University Yong-san Hospital were screened over a 1-year period. A total of 100 eligible patients who agreed to participate in this study were consecutively included. The exclusion criteria were as follows: American Society of Anesthesiologists status III-V; history of an upper respiratory infection within the previous 4 weeks; having neurologic symptoms causing poor cooperation during endoscopy; psychological disorders; respiratory diseases requiring oxygen therapy, such as bronchial asthma, chronic obstructive respiratory disease, or sleep apnea; pregnancy; Child-Pugh class B or C cirrhosis; chronic kidney disease; receiving treatment with psychoactive medications; an illicit drug user or a heavy alcoholics; a history of allergic reaction to drugs to be administered; other severe medical conditions not suitable for sedative endoscopy; undergoing endoscopic procedures in an emergency setting. In addition, we also excluded patients taking medications which can interact with drugs in the benzodiazepine class, or midazolam specifically, such as erythromycin, verapamil, diltiazem, itraconazole, and ketoconazole. The study protocol was approved by the Institutional Review Board of Chung-Ang University College of Medicine (No. 2006-013-08-1). This study was performed in accordance with the guidelines and regulations of the above mentioned Ethics Committees, and written informed consent was obtained from all study participants.

### Sedation protocol

As premedication for endoscopic examination, the patients were given 5 mg of cimetropium bromide by intramuscular injection, 10 mL of simethicone bromide orally 20 minutes before the procedure. For topical anesthesia of pharynx, the patients were asked to gargle with 10 mL of lidocaine viscous oral solution 2% for 5 minutes before swallowing it. The patients received oxygen insufflation at a rate of 2 L/min via nasal cannula throughout the procedure. Sedation was performed with intravenous bolus midazolam at a dose of 0.06 mg/kg. Prior to the administration of midazolam, consciousness was assessed in all the patients. To achieve conscious sedation in the patients, the target level of sedation was set to moderate degree, which means that patients can respond purposefully to verbal commands, either alone or accompanied by light tactile stimulation.

### Patient monitoring and data collection

Blood pressure, heart rate, respiration rate, arterial oxygen saturation, and the electrocardiogram (DS-5100E; Fukuda, Denshi, Japan) were monitored continuously throughout the procedure. For measurement of plasma midazolam concentration and genotype testing, 5 mL of venous blood was collected 20 minutes after the administration of midazolam. The blood was stored in a vacuumed ethylenediaminetetraacetic acid tube at −30 °C. Upper GI endoscopy was performed by one experienced gastroenterologist (J.K.) in a standardized environment using videoendoscopy equipment (EVIS 240 Q; Olympus, Tokyo, Japan).

To monitor and assess the level of sedation, the Ramsay Sedation Scale (Table 1) was recorded 2 minutes after the administration of midazolam. Adverse reactions such as paradoxical response and anterograde amnesia were also evaluated. The satisfaction level of the patients and endoscopists after the procedure were measured using the verbal numerical rating scale (VNRS). To assess recovery from conscious sedation during the recovery period after finishing the procedure, monitoring using the Modified Aldrete Scoring System was conducted. This score was assessed at three time points: at arrival in the recovery room after finishing the procedure, at 5 and 15 minutes after the first assessment. An independent observer was responsible for the monitoring, which included evaluation of the level of consciousness, readouts of other vital signs, collection of data regarding drugs and doses administered, use of benzodiazepine antagonists (flumazenil), and occurrence of cardiorespiratory adverse events such as hypoxemia (defined as the peripheral capillary oxygen saturation <90% for >30 seconds after application of the jaw thrust maneuver), hypotension (defined as ≥20% reduction in systolic or diastolic blood pressure), and bradycardia (heart rate <50 bpm).

**Table 1:** Ramsay Sedation Scale.

Ramsay Sedation Assessment	Score
Anxious and agitated or restless, or both	1
Co-operative, oriented, and calm	2
Responsive to commands only	3
Exhibiting brisk response to light glabellar tap or loud auditory stimulus	4
Exhibiting a sluggish response to light glabellar tap or loud auditory stimulus	5
Unresponsive	6

The same observer also reported any other adverse events that occurred secondary to sedation. The paradoxical response was defined as a condition where midazolam precipitated hostility, rage, and physical violence, necessitating the restraint of the patient until the effects diminished spontaneously. Retrograde amnesia was evaluated by delayed recall of three easily recognizable, commonly used words. Patients were asked to memorize each word at the time of verbal and visual presentation right before the intravenous administration of midazolam. Memorization and verbal recall were confirmed immediately after the initial presentation of the words. To minimize the effect of decreased cognitive function by midazolam, the observer assessed patients’ recall of words only after the patient was leaving the recovery room. If the patient failed to remember any of the three words, retrograde amnesia was considered present. The patients were discharged once they had achieved ≥9 on the Modified Aldrete Scoring System and had reported no pain or any other type of discomfort. Every patient completed a satisfaction questionnaire before leaving the facility. The observer responsible for the monitoring contacted the patients to administer a questionnaire that evaluated patients’ satisfaction with the procedure, adverse events, and the resumption of domestic activities. Patient satisfaction was evaluated with the VNRS system (from 0; least satisfied to 10; most satisfied). Similarly, the VNRS was also applied to the endoscopists to assess the level of satisfaction regarding the patient cooperation and ease of endoscopic procedures.

### DNA extraction

Blood samples were centrifuged for genotype testing and genomic DNA was extracted by QIAamp DNA Mini kit (Qiagen Inc., Valencia, CA, USA) using the leukocyte layer. Blood samples were then centrifuged at 2500 rpm for 10 minutes followed by separation of the leukocyte layer. In the leukocyte layer, 200 μl was collected, mixed with 20 μl QIAGEN protease and 200 μl Buffer AL, and then shaken in a vortex mixer for 15 seconds. After incubation at 56 °C for 10 minutes, the mixture was centrifuged. A total of 200 μl of 96–100% ethanol was added and the sample was vortexed again for 15 seconds, after which it was centrifuged again at 2500 rpm for 10 minutes. The mixture was transferred into a QIAamp Spin Column and centrifuged at 13000 rpm for 1 minute. The DNA was then extracted according to the manufacturer’s recommendation. The A260/A280 ratio of the extracted DNA samples was 1.7–1.9, confirming that they were relatively pure.

### Genotype analysis

The method of Lindberg *et al*.^9^ was adopted for DNA amplification by polymerase chain reaction (PCR). The total PCR reagent reaction volume was 25 μl, comprising 2 μl g-DNA, 0.1 μl Taq polymerase (Takara, Japan), 2 μl (100 µM) deoxynucleotide, 2.5 μl 10 × buffer, 17.4 μl AC D/W, and 0.5 μl sense and antisense primers. The PCR conditions were as follows: 5 minutes of pre-denaturation at 94 °C, followed by 30 cycles of 30 seconds of denaturation at 94 °C, 56 annealing at 60 °C for 30 seconds, synthesis for 1 minute at 72 °C, and extension for 10 minutes at 72 °C. The genotypes of *MDR1* 1236C > T (assay ID: C__7586662_10), ABCG2 421C > A (assay ID: C__15854163_70), and ABCC2 1249G > A (assay ID: C__22272980_20) single nucleotide polymorphism (SNP) were analyzed by the established TaqMan Genotyping Assays (Applied Biosystems, Foster City, CA, USA).

### Statistical analysis

Data were expressed as number or percentage (%) and analyzed statistically using the Statistical Package for Social Sciences program (SPSS v 18.0, IBM Corp., Armonk, NY, USA). Alleles of modified SNP and genotype frequency were evaluated by Chi-square test with deviation from Hardy-Weinberg equilibrium. Correlation between genetic polymorphism and plasma concentration of midazolam and 1-hydroxymidazolam was analyzed with Student’s t-test. A chi-squared test or Fisher’s exact test was used to analyze the correlation between genetic polymorphism and the grade of sedation assessed with the Ramsay Sedation Scale, and adverse events after administering midazolam. Results were considered statistically significant at *P* < 0.05.

## Results

### Baseline demographics and clinical characteristics

A total of 100 patients participated in the study, and their baseline characteristics are shown in Table 2. The mean values of body weight, height, and body mass index were 63.8 ± 10.9 kg, 164 ± 8.0 cm, and 23.57 ± 3.41 kg/m^2^, respectively. Following midazolam administration, 13 patients (13%) exhibited a paradoxical response and 32 patients (32%) showed retrograde amnesia. One patient complained of drowsiness after midazolam administration. During endoscopy, two patients experienced hypoxemia and three patients exhibited hypotension, which were temporary and quickly recovered with conservative management. The mean Ramsay Sedation Score was 2.8 ± 0.1. The mean Modified Aldrete Score was 9.02 ± 0.75, 8.99 ± 0.89, and 9.07 ± 0.84 at 0, 5, and 15 minutes after the arrival in the recovery room, respectively. The satisfaction scores evaluated with VNRS (0–10) for the patients and the endoscopist were 8.59 ± 1.91 and 8.25 ± 2.11, respectively.

**Table 2:** Baseline characteristics of study subjects.

Variables	Participants (n = 100)
Age (years), mean ± SD	43.26 ± 13.87
Sex, male	51 (51)
Height (cm), mean ± SD	164.53 ± 8.06
Body weight (kg), mean ± SD	63.88 ± 10.98
Body mass index (kg/m^2^), mean ± SD	23.57 ± 3.41
Paradoxical response	13 (13)
Retrograde amnesia	32/99 (32.3)
Modified Aldrete score (0 min)	9.02 ± 0.75
Modified Aldrete score (5 min)	8.99 ± 0.89
Modified Aldrete score (15 min)	9.07 ± 0.84
Plasma concentration of midazolam (ng/mL)	71.07 ± 18.79
Plasma concentration of 1-hydroxymidazolam (ng/mL)	31.24 ± 3.32

The values are shown as mean ± SD or number (%). SD, standard deviation.

### Biochemical and genetic profiles of the patients

**Figure 1:**
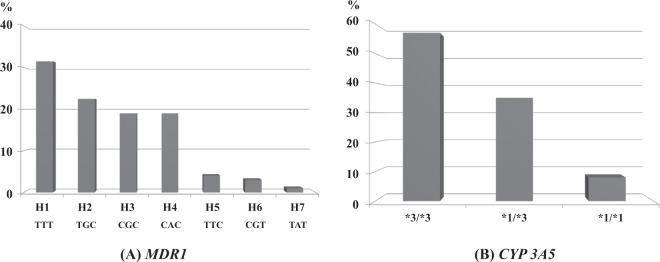
Frequency of *MDR1* and *CYP3A5* genetic polymorphism. The frequency of *MDR1* and *CYP3A5* genetic polymorphism among 100 subjects showed that TTT (H1) was the highest whereas TAT (H7) was the lowest in *MDR1* haplotypes. (**A**) CYP3A5 *3/*3 was the highest whereas *1/*1 was the lowest in *CYP 3A5* (**B**).

The mean plasma concentration of midazolam and 1-hydroxymidazolam was 71.0 ± 18.8 ng/mL and 31.2 ± 13.3 ng/mL, respectively. All patients were genotyped for genetic variants in *MDR1* and *CYP3A5*. For haplotype estimation in five genotypes (1236C > T, 3435C > T, 2677G > T, 2677G > A, and 2677G > T/A) in the *MDR1* gene, TTT, TGC, CGC, CAC, TTC, CGT, and TAT were predominately generated. The frequency of each genetic variation is shown in Fig. 1A, and TTT haplotype was the most frequently observed. With regard to *CYP3A5*, the 3*/3* type was the most frequently detected (Fig. 1B).

### Correlation between genetic polymorphism and plasma midazolam concentration

**Figure 2:**
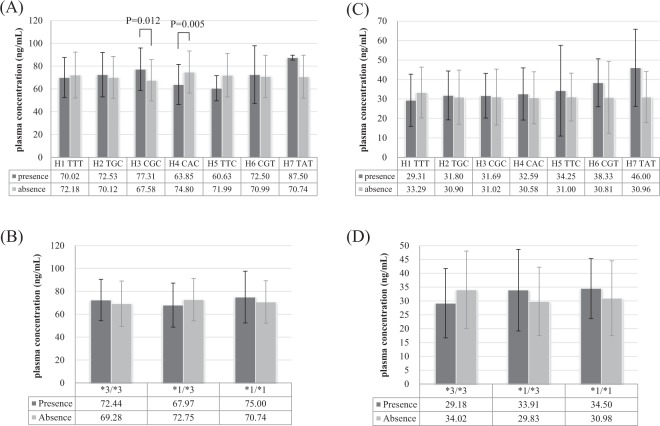
Correlation between genetic polymorphism and plasma concentration of midazolam and 1-hydroxymidazolam (ng/mL). *MDR1* and the concentration of midazolam. (**A**) *CYP3A5* and the concentration of midazolam. (**B**) *MDR1* and the concentration of 1-hydroxymidazolam. (**C**) *CYP3A5* and the concentration of 1-hydroxymidazolam (**D**).

The plasma concentration of midazolam in patients with CGC haplotype of *MDR1* was greater than that in patients with other haplotypes (*P* = 0.012). On the contrary, plasma concentration of midazolam in patients with CAC haplotype was lower than that in patients with other haplotypes (*P* = 0.005, Fig. 2A). However, the genetic polymorphism of *CYP3A5* was not associated with the concentration of midazolam (Fig. 2B). Neither genetic polymorphism of *MDR1* nor *CYP3A5* correlated with the concentration of 1-hydroxymidazolam (Fig. 2C,D).

### Correlation between genetic polymorphism and sedative effect

As shown in Table 3, CGT haplotype of *MDR1* was significantly associated with sedation grade after midazolam administration (*P* = 0.042). Meanwhile, this association was not detected with genetic polymorphism of *CYP3A5*. With regards to adverse events, there was no association between genetic polymorphism of *MDR1* or *CYP3A5* and paradoxical response or retrograde amnesia (Table 4).

**Table 3:** Genetic polymorphism and grade of sedation: (A) *MDR1*, (B) *CYP3A5*.

	Ramsay score ≤3 (n = 85)		Ramsay score ≥4 (n = 15)		*P*-value
(**A**) ***MDR1***					
H1 TTT	45	(52.9%)	6	(40.0%)	0.355
H2 TGC	33	(38.8%)	7	(46.7%)	0.568
H3 CGC	32	(37.6%)	4	(26.7%)	0.414
H4 CAC	27	(31.8%)	7	(46.7%)	0.261
H5 TTC	7	(8.2%)	1	(6.7%)	1.000
H6 CGT	3	(3.5%)	3	(20.0%)	0.042
H7 TAT	2	(2.4%)	0	(0%)	1.000
(**B**) ***CYP3A5***					
*3/*3	49	(57.6%)	8	(53.3%)	0.071
*1/*3	28	(32.9%)	7	(46.7%)	0.145
*1/*1	8	(9.4%)	0	(0%)	0.476

**Table 4:** Genetic polymorphism and paradoxical response and retrograde amnesia: (A) *MDR1*, (B) *CYP3A5*.

	Paradoxical response (+) (n = 13)		Paradoxical response (−) (n = 87)		*P*-value	Retrograde amnesia (+) (n = 32)		Retrograde amnesia (−) (n = 67)		*P*-value
**(A)** ***MDR1***										
H1 TTT	9	(69.2%)	42	(48.3%)	0.159	14	(43.8%)	37	(55.2%)	0.285
H2 TGC	4	(30.8%)	36	(41.4%)	0.466	14	(43.8%)	26	(38.8%)	0.639
H3 CGC	4	(30.8%)	32	(36.8%)	0.765	12	(37.5%)	23	(34.3%)	0.758
H4 CAC	4	(30.8%)	30	(34.5%)	1.000	12	(37.5%)	22	(32.8%)	0.648
H5 TTC	0	(0%)	8	(9.2%)	0.592	3	(9.4%)	5	(7.5%)	0.711
H6 CGT	1	(7.7%)	5	(5.7%)	0.576	1	(3.1%)	4	(6.0%)	1.000
H7 TAT	0	(0%)	2	(2.3%)	1.000	1	(3.1%)	1	(1.5%)	0.544
(**B**) ***CYP3A5***										
*3/*3	7	(53.8%)	50	(57.5%)	0.805	18	(56.2%)	38	(56.7%)	0.955
*1/*3	4	(30.8%)	31	(35.6%)	1.000	10	(31.2%)	25	(37.3%)	0.555
*1/*1	2	(15.4%)	6	(6.9%)	0.278	4	(12.5%)	4	(6.0%)	0.269

## Discussion

The present study examined the association between the genetic polymorphism of genes involved in midazolam metabolism and various responses to midazolam in conscious sedation during UGI endoscopy. We observed patient responses after administrating midazolam and assessed the association with *MDR1* and *CYP3A5* gene polymorphism. The presence of CGC or CAC haplotype of *MDR1* was significantly associated with the plasma concentration of midazolam. With regard to the sedative effect, CGT haplotype of *MDR1* was associated with higher sedation grade following midazolam administration. In contrast, there was no direct correlation between genetic polymorphism of *MDR1* or *CYP3A5* and adverse reactions such as paradoxical response and retrograde amnesia.

The main roles of sedation in endoscopy are to alleviate patients’ anxiety and discomfort, improve the outcome by enhancing patient tolerance to the procedure, and reduce unpleasant memories related to the procedure. From the endoscopist’s perspective, sedation contributes to the successful completion of endoscopic procedures and improves procedural outcomes^10^. Midazolam has become a popular sedative for conscious sedation in endoscopy due to its favorable properties, such as rapid onset and short duration of action, and profound anterograde amnestic effects^11^. The action of midazolam is achieved by the potentiation of neural inhibition mediated by GABA, an inhibitory neurotransmitter, producing hypnotic activity, sedation, anxiolytic activity, amnesia, anticonvulsant activity, and muscle-relaxant activity^12^.

Although midazolam has many advantageous characteristics, a number of adverse reactions such as over-sedation and paradoxical excitation can occur^2^. It is obvious that respiratory depression and cardiovascular effects are very well known side effects of sedation which may have serious consequences, and can be objectively assessed with continuous monitoring of vital signs. On the other hand, adverse events such as paradoxical response or retrograde amnesia are not easy outcomes to assess objectively. Nevertheless, this study intended to see the effect of genetic polymorphism on these relatively under-investigated outcomes. While the causes and risk factors of paradoxical response or retrograde amnesia are multifactorial and not clearly revealed yet, there were some studies suggesting the effect of genetic background in paradoxical response^8^. Indeed, in this study, 13 patients exhibited a paradoxical response and 32 had retrograde amnesia after they were administered 0.06 mg/kg of midazolam intravenously. There were also three hypotension and two hypoxic events, which were all transient and resolved spontaneously or by simple airway maneuvers including jaw lifting, airway maintenance, and oxygen supplement only. The number of these patients with cardiorespiratory events was too small for additional subgroup analysis. It has been suggested that the personality of the individual, genetic factors, gender, degree of patient apprehensiveness, and chronic alcoholism may be predictive factors of adverse reactions^13^. While the mechanism behind these reactions is largely unknown, these adverse reactions have been suggested to be dose-dependent. However, these adverse events do not always occur in the same dose-dependent way, and are often unpredictable in many of the cases. One of possible explanations for this inconsistency and unpredictability in occurrence of adverse reactions might be the individual differences in metabolism of midazolam. Midazolam is oxidized initially in the liver and undergoes oxidative metabolism catalyzed by the CYP3A subfamily to the metabolite, 1-hydroxymidazolam^14^. Human CYP3A is involved in the oxidation of numerous drugs^15^, and many of the studied CYP3A substrates are transported by P-glycoprotein (P-gp), which is encoded by the *MDR1* gene^16^. Consequently, polymorphism of the *MDR1* gene can cause reduced response, refractoriness or resistance to drugs^17^. This individual variability might subsequently result in differences in the level of sedation and adverse reactions of sedative medication. Based on this, it can be assumed that a patient’s response to midazolam may be related with *MDR1* gene polymorphism. Therefore, this study investigated the quality of sedation related to midazolam administration from a viewpoint of genetic polymorphism.

We found that the presence of CGC haplotype and absence of CAC haplotype in the *MDR1* gene was both associated with increased plasma concentration of midazolam. These haplotypes may be linked to the P-gp substrate that reacts with midazolam^5^. Due to the association between P-gp and CYP3A, that reduces drug absorption, the metabolism of midazolam to 1-hydroxymidazolam should be modulated by P-gp^18^. P-gp inhibition resulted in a modest increase in midazolam metabolism at higher midazolam concentrations^18^. However, the concentration of midazolam did not correlate with adverse reactions including paradoxical response and retrograde amnesia. Since adverse reactions occur in dose-dependent manner^7^, another factor besides plasma midazolam concentration may be attributable to occurrence of these adverse reactions.

CGT haplotype of *MDR1* gene was associated with higher sedation grade after administrating midazolam (*P* = 0.042). This suggests that the CGT haplotype may be associated with the binding of midazolam to the P-gp substrate. Therefore, variations in the phenotypic activity of P-gp due to genetic polymorphisms or competitive inhibition may determine a patient’s susceptibility to sedative effects and side effects of midazolam.

However, neither *MDR1* nor *CYP3A5* genetic polymorphism correlated with a paradoxical response after administrating midazolam in the present study. Although the exact mechanism of such paradoxical reactions is unknown, several risk factors for this reaction have been reported including midazolam dose, age, gender, psychological background, alcohol abuse, and genetic background^7^. In particular, genetic variability in the benzodiazepine receptors of GABA_A_ channels, resulting from the multiple allelic forms, may lead to abnormal pharmacodynamic responses in some patients^19^. Although the mechanism behind the midazolam and retrograde amnesia relationship is unknown, retrograde amnesia post administration of midazolam is dose dependent^20^. However, there was no significant association between genetic polymorphism and retrograde amnesia following administrating midazolam in the present study. These results suggest that paradoxical response and retrograde amnesia may be a P-gp-independent reaction.

There were a few limitations in this study. First, some patients with insufficient sedation might have shown aggressiveness, violent behavior, or hyperactivity during the procedures. However, it is impossible to perfectly distinguish between insufficient sedation and paradoxical response. Besides, the mean Ramsay Sedation Score was 2.8 in the present study. Moderate sedation, regarded as the appropriate state in conscious sedation, corresponds to a score of three on the Ramsay Sedation Scale (exhibiting brisk response to light glabellar tap or loud auditory stimulus). The satisfaction scores reported by patients and endoscopists were also consistently high. These results indicate that the quality of sedation in this study was generally adequate from the viewpoint of both the patients and endoscopists. Second, it was difficult to confirm the correlation among genetic polymorphism and adverse reactions related with midazolam in relatively small number of patients. In addition, it was difficult to completely rule out the possibility of transient cognitive impairment induced by midazolam might have affected the test results for retrograde amnesia. To minimize the possible effect of impaired cognitive function, the test was done when the patients were leaving the recovery room after confirming the modified Aldrete Score met the criteria. Nevertheless, the true frequency of retrograde amnesia could be lower than the results of this study. Third, as this study investigated the pharmacologic and clinical perspectives of midazolam only, one should be cautious in generalizing the results to the clinical circumstances where mixed sedative agents are concurrently administered. Nevertheless, midazolam has been commonly used as a single sedative agent and still being frequently used in sedation endoscopy in many countries, which makes the results of this study valuable. Fourth, we used Ramsay sedation scale for monitoring the sedation depth during the procedure, which was further dichotomized into two groups in the analysis. This approach to the evaluation and assessment of sedation might be regarded rather crude, compared to EEG monitoring or a combination of clinical sedation scales. However, these advanced monitoring methods are neither easily applicable nor routinely used in diagnostic upper endoscopy sessions. Despite the objectivity issue, the Ramsay sedation scale still has advantages of easy applicability in real clinical practice.

In conclusion, genetic polymorphism in *MDR1* gene was associated with plasma midazolam concentration and sedation grade after midazolam administration. Although genetic polymorphism was not associated with adverse reactions, patients’ responses and conscious states varied following the administration of midazolam. Therefore, close monitoring until discharge is mandatory in upper GI endoscopy under sedation.

## Data Availability

All data generated or analyzed during this study are included in this published article.
